# Assessment of Information to Substantiate a Health Claim on the Prevention of Prostate Cancer by Lignans

**DOI:** 10.3390/nu2020099

**Published:** 2010-01-28

**Authors:** Niina M. Saarinen, Juhani Tuominen, Liisa Pylkkänen, Risto Santti

**Affiliations:** 1 Functional Foods Forum, University of Turku, Turku, 20014, Finland;; 2 Department of Statistics, University of Turku, Turku, 20014, Finland; Email: juhani.tuominen@utu.fi; 3 Department of Oncology, University of Turku, Turku, 20014, Finland; 4 Medical School, University of Tampere, Tampere, 33014, Finland; Email: liisa.pylkkanen@uta.fi; 5 Institute of Biomedicine, University of Turku, Turku, 20014, Finland; Email: risto.santti@pp.inet.fi

**Keywords:** lignan, phytoestrogen, diet, prostate cancer, health claim, surrogate biomarker

## Abstract

Lignans and their *in vivo* metabolites, especially enterolactone (ENL), have attracted substantial interest as potential chemopreventive agents for prostate cancer. Preclinical and clinical interventions performed with lignan-rich flaxseed that use surrogate biomarkers as endpoints suggest that lignans may attenuate prostate carcinogenesis in individuals with increased risk or with diagnosed cancer. No unequivocal prostate cancer risk reduction has been found for lignans in epidemiological studies, suggesting that lignan concentrations found in populations consuming a regular non-supplemented diet are not chemopreventive in prostate cancer. Presumably, the main obstacles in assessing the efficacy of food lignans is limited knowledge of the serum and tissue lignan concentrations required for the putative prevention. Further clinical studies performed with the purified compounds are required to substantiate a health claim.

## 1. Introduction

According to the 2005 WHO Global Infobase prostate cancer is the most common cancer and among the three leading causes of the cancer-related deaths in men in the North America and northern European countries. The lifetime risk of men being diagnosed with prostate cancer exceeds 1 in 10 in several countries. The severity is highly variable, from indolent disease to aggressive cancer, which may lead to death even within months. The causes of prostate cancer are not known. The genetic factors (family background) contribute to the risk and the risk is increasing with age. At present, there are no known preventive means to reduce the risk for clinical prostate cancer.

There are considerable variations in the prostate cancer incidence and mortality rates between different countries and ethnic groups. The increasing incidence rates among the immigrants from Asian countries to Europe and North America are associated with the change of the traditional Asian life-style and diet to the Western life-style and diet. This suggests that environmental, particularly dietary factors significantly influence the development and progression of prostate cancer [1,2]. 

Pathogenesis of prostate cancer starts at the age of 30–40 years giving a distinct opportunity for prevention through dietary modifications. The most intense prevention interests by diet have focused on vitamin E, selenium, lycopene, vitamin D and the analogues, and isoflavones. However, recent studies exploring the effects of these dietary components on prostate cancer incidence rates have been disappointing. In the SELECT study, selenium or vitamin E, alone or in combination, did not prevent prostate cancer [3]. Neither vitamin E nor vitamin C supplementation reduced the risk of prostate or any other cancer in The Physicians’ health study II [4] and blood lycopene concentrations of men with prostate cancer were not different from those in men without signs of prostate cancer [5]. These findings suggest that other components in diet are likely to account for the preventive effect against prostate cancer or that single agents, even in combinations, may be an ineffective approach to primary prevention of prostate cancer

**Figure 1:**
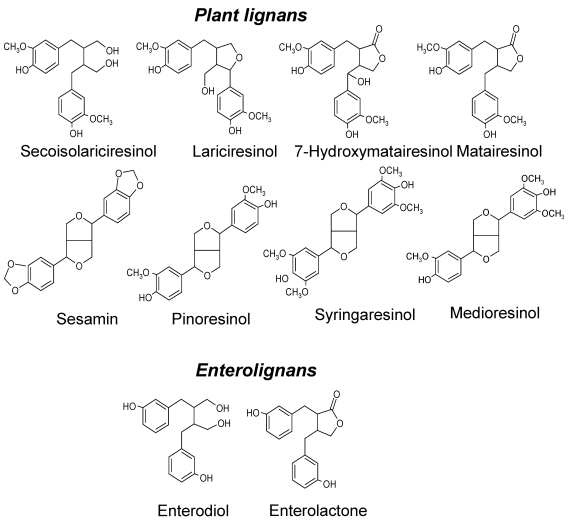
Chemical structures of most abundant dietary plant lignans and their enterolignan metabolites.

In Western diet, lignans and their *in vivo* metabolites, especially enterolactone (ENL), have gained substantial interest as potential chemopreventive agents. The most abundant plant lignans in foods are secoisolariciresinol, lariciresinol, pinoresinol, syringaresinol, medioresinol, 7-hydroxymatairesinol (HMR), and sesamin [6,7]. When ingested, all of these plant lignans can be absorbed as such [8,9,10] and converted further to enterolignans, e.g., enterodiol and ENL, by intestinal bacteria [11,12] (Figure 1). The richest dietary sources of plant lignans are flaxseed and sesame seed containing mainly secoisolariciresinol diglucoside and sesamin, respectively. In addition, high consumption of whole grains (such as rye), vegetables, and fruits may provide a significant portion of the ingested lignans although the quantities are low compared to flaxseed and sesame seed. 

In this chapter, we summarize the findings of lignan-rich diets, isolated dietary lignans, and ENL on prostate cancer in epidemiological studies, clinical interventions and preclinical models. The opportunities to use surrogate biomarkers in clinical intervention trials in healthy men and prostate cancer patients are discussed and the clinical studies performed with lignan-rich flaxseed are summarized. Further, the currently available data of lignans to substantiate a health claim on the prostate cancer risk reduction is discussed according to the guidelines and opinions given by European Food and Safety Authority (EFSA). 

## 2. Epidemiological Studies on Lignans and Prostate Cancer Risk

Until now, four epidemiological case-control studies and seven studies with prospective designs on the association of lignans with the risk of prostate cancer have been published (Table 1). In the studies, the risk has been correlated with serum or plasma ENL concentration, urinary ENL excretion, or intake of specific dietary lignans. In nine studies out of eleven, no significant correlation between serum, plasma or urine ENL or dietary lignan intake and prostate cancer risk or progression has been observed (Table 1). In the most recent one [13], the plasma concentrations of ENL and enterodiol were evaluated in relation to risk of the subsequent prostate cancer in a case-control study nested in the European Prospective Investigation into Cancer and Nutrition. No statistically significant associations were observed for ENL or enterodiol to overall risk of prostate cancer.

In the studies relating to dietary intake, the mean daily total lignan intake varied from 0.5 mg [2,14,15] to 3 mg [15], likely depending whether the intake of two or six lignans were assessed. In all studies investigating circulating ENL concentrations, the mean serum and plasma ENL concentration were below 20 nM [13,16,17,18,19,20]. One hypothesis is that low levels of lignans in diet may explain the small differences in serum and urine ENL concentrations as well as in lignan intakes between the compared population quartiles. This may impede finding of associations. Moreover, within an individual, the plant lignan intakes and consequently serum enterolignan concentrations vary and a single ENL measurement data does not necessarily reflect the exposure to plant lignans over an extended period of time [21]. Further, serum concentrations of ENL depend not only on dietary intake of plant lignans but also e.g., on their biotransformation by gut microflora, absorption and excretion, and the amount of dietary fat consumed [8,10,11]. 

**Table 1:** Epidemiological studies on the association between lignans and prostate cancer risk.

*Type of Study*	*Study population*	*Outcome*	*Ref.*
Nested Case-Control	Multiethnic cohort in Hawaii and Califormia, 249 cases, 404 controls	No association between urinary ENL excretion and prostate cancer risk.	[68]
Nested Case-Control	EPIC cohort, 950 cases, 1042 controls	No association between plasma enterodiol or ENL and prostate cancer risk.	[13]
Nested Case-Control	EPIC-Norfolk cohort, 193 cases, 828 controls	No association between prostate cancer risk and total serum lignans or enterodiol or ENL.	[20]
Prospective	British men, 191 prostate cancer patients, 71 progressed diseases during median of 2.5 years follow-up	No association of baseline urinary ENL levels with time to disease progression. No association between adverse histology on repeat biopsy or PSA velocity and urinary ENL.	[69]
Case-Control	Scottish men, 50-74 y, 433 cases, 483 controls	Inverse association with increased serum concentrations of ENL and prostate cancer risk.	[19]
Case-Control	Swedish men, 35-79 y, 1499 cases, 1130 controls; 209 cases, 214 controls for assessment of serum enterolactone	No association between dietary intake of total or individual lignans or isoflavonoids and risk of prostate cancer. Intermediate serum levels of ENL were associated with a decreased risk of prostate cancer.	[15]
Case-Control	Men in Western New York, 433 cases, 538 controls	Reduced risk of prostate cancer in men in the highest quartile of intake of total lignan precursors* compared with men in the lowest quartile of intake.	[2]
Prospective	Swedish men, 265 cases, 525 controls. Mean follow-up 5 years	No significant association between quartiles of plasma ENL and risk of prostate cancer.	[18]
Nested Case-Control	Finnish male smokers, 50-69 y, 214 cases, 214 controls	No association between serum ENL concentrations and prostate cancer risk.	[17]
Case-Control	Caucasian men in Texas, 83 cases, 107 controls	No association between high intake of lignans** and prostate cancer risk.	[14]
Nested Case-Control	Finnish, Norwegian and Swedish men, 794 cases, 2550 controls	No association between serum ENL concentrations and prostate cancer risk in full study group or in national groups.	[16]

* secoisolariciresinol and matairesinol, ** secoisolariciresinol, matairesinol, lariciresinol, pino-resinol, syringaresinol, and medioresinol

These epidemiological findings strongly suggest that lignans at levels found in populations consuming a regular non-supplemented diet are not chemopreventive in prostate cancer. Until now, the lowest observable effect level for any lignan in prostate cancer is not known. In the preclinical and clinical studies performed so far with the lignan-rich foods and diets, the serum or tissue lignan levels are not reported. However, separate lignan metabolism studies performed in animals and humans suggest that the supplementations used in the intervention studies result in serum concentrations of several hundred nanomolar up to micromolar [8,9,12]. Daily consumption of whole grain fiber, fruit or vegetable increase serum ENL levels up to 50-100 nM [22,23]. Ingestion of a single 50 g dose of sesame seeds has been reported to increase serum enterodiol and ENL concentrations above 1 μM [9]. In all intervention studies performed with flaxseed, a significant increase in serum ENL levels has been measured [24]. The highest serum ENL concentrations (>300nM) have been reported following ingestion of flaxseed lignan extracts [25,26]. Thus, the negative findings of lignans against prostate cancer in epidemiological studies may indicate inefficiency of low dietary lignan exposures over extended period of time rather than the inefficiency of lignan supplementation in general.

## 3. Preclinical *in vivo* Studies of Lignans and Lignan-Rich Foods

Prostate cancer can be considered as a spectrum of diseases from androgen-responsive to androgen-nonresponsive and from local to metastatic disease with distinct characteristics and origin. Thus, it is highly unlikely that any single experimental model would extensively depict the disease. However, the variety of *in vivo* prostate cancer models currently available offers opportunities for research on the role of lignans and lignan-rich diets in different stages of carcinogenesis. Until now, only two studies have been published. 

In the first study performed, the effects of dietary flaxseed were studied in the transgenic adenocarcinoma of mouse prostate (TRAMP) model [27]. The TRAMP mice harbor a rat probasin promoter that drives prostate-specific epithelial expression of transgene SV40 T-antigen at puberty [28,29]. The viral transgene drives transformation of normal epithelium first to prostatic intraepithelial neoplasia (PIN) with low- and high-grade characteristics and further to androgen-dependent and -independent prostate adenocarcinomas and distant metastases [30]. In the TRAMP model with C57BL/6 background, 5% flaxseed added to AIN-76 diet and started at the time of puberty did not have significant effect on prostate cancer incidence compared to an isocaloric control diet with equivalent percentages of protein, carbohydrate, and fat [27]. However, the flaxseed diet inhibited the growth and progression of the established cancers, and after 30 wk treatment, the tumors in the flaxseed group were less aggressive in phenotype than those in the control group [27]. Moreover, flaxseed supplementation increased cellular apoptosis and decreased proliferation in the prostate [27]. 

In the second study, HMR, a purified plant lignan added to AIN-93G diet, was tested in *s.c.* human LNCaP xenografts in athymic mice [22]. LNCaP cells are androgen-responsive PSA producing prostate cancer cells originally isolated from a lymph node metastasis [31]. The implanted cells can be grown as *s.c.* or orthotopic xenografts in athymic mice and rats. Phenotypically, the formed xenograft tumors represent advanced prostate cancer but the tumors seldom metastasize. In the study, 0.15% and 0.3% HMR supplemented AIN-93 diets (resulting in daily intake of 13.4 and 25.8 mg/kg, correspondingly) or control AIN-93 diet were fed to mice starting 3 days after *s.c.* injections of LNCaP cell with BD Matrigel ^TM^ and basic fibroblast growth factor. The tumor take rate (number of injected sites developing into tumors) and the growth of the tumors were used as surrogate endpoints. At the end of the 9-week study, the mice on the HMR-supplemented diets had lower tumor take rate, smaller tumor volumes, and relatively more non-growing tumors than the control mice. This may be explained by significantly decreased tumor cell proliferation rate, as well as increased rate of apoptosis. HMR had no effect on the weight of ventral or dorsolateral lobes of prostate. 

As described above, the findings in the experiments with lignan-rich diet containing flaxseed and HMR are in agreement with the putative significance of dietary lignans in prostate carcinogenesis. To our knowledge, HMR is the only dietary lignan tested in an animal model of prostate cancer. So far, no studies performed with the most abundant enterolignan ENL have been reported. For possible chemoprevention by dietary compounds, we need to know the significance of lignans, *i.e.*, which compounds are bioactive, and which doses are needed for the desired effect. Otherwise, it is impossible to identify which foods or food components would fulfill the requirements. As an example, the composition of flaxseed may vary considerably depending on differences in cultivation conditions [32] and processing [33]. Moreover, it is crucial to confirm the efficacy of lignans in the final product. Therefore, systematic, stepwise approach is unavoidable when investigating the role of dietary components in cancer. 

## 4. Potential Mechanisms of Action of Lignans in Prostate Cancer

There is a need to better understand the actions by which lignans could have anticarcinogenic effects. Enterolignans, particularly ENL, have been suggested to exert the anticarcinogenic activity of dietary lignans [24]. An antiproliferative action of ENL has been demonstrated in androgen-responsive LNCaP cells representing early stages of prostate cancer and in androgen-nonresponsive PC-3 and DU-145 cells representing early and later stage of prostate carcinogenesis, correspondingly [34]. Inhibition of LNCaP cells by ENL has been documented to occur through altered expression of cell cycle associated genes [35]. In PC-3 cells, ENL inhibits insulin like growth factor (IGF-1) –induced cell proliferation and migration and attenuates IGF receptor (IGF-1R) mediated cell signaling [36].

Enterolignans and their dietary precursors have constantly been shown to increase cancer cell apoptosis *in vivo* [37,38,39], which may explain at least in part their anticarcinogenic potency. These findings have been verified in cell cultures *in vitro*, where the induction of apoptosis in LNCaP cells by ENL has been shown to occur through mitochondrial-mediated, caspase-dependent pathway [40]. Moreover, reduced expression of survivin (BIRC5) in ENL treated LNCaP cells further supports the caspase –mediated apoptosis pathways as one of the targets for the lignan action in prostate cancer [35]. Recent findings indicate that ENL sensitizes prostate cancer cells to tumor necrosis factor-related apoptosis-inducing ligand (TRAIL)-induced apoptosis [41]. High lignan concentrations (Ź10 μM) clearly exceeding those reported in human serum have been used in the majority of the *in vitro* experiments and the relevance of the findings remains thus unclear. Moreover, there is limited data available on the effects of dietary plant lignans on prostate cancer cells although many of those are absorbed [8]. The plant lignans may also be biologically active. For instance PSA expression is reduced and TRAIL-induced apoptosis increased in LNCaP cells by secoisolariciresinol and matairesinol, correspondingly [41,42].

Plant and enterolignans are commonly classified as phytoestrogens. The pertinent question is the estrogenicity or antiestrogenicity of different lignans. Several studies have shown that ENL stimulates the proliferation of estrogen-dependent cancer cells [43] suggesting that ENL is an estrogen. The cells most often used are breast cancer cells (MCF-7 and T-47D) expressing both estrogen receptors ERα and ERβ at various ratios. Above 1 μM ENL concentrations have been used to demonstrate an increased proliferation, and no antagonistic action has been observed when the cells were co-treated with estradiol and ENL. Surprisingly, Cosentino and coworkers [44] have demonstrated an estrogen-like activity for ENL at picomolar concentration by using the MCF-7 cells entering to the S phase of the cell cycle as a parameter. 

The role of estrogens in the prostate is complex. There is accumulating evidence for the essential role of estrogens in malignant growth of the prostate [45,46]. On the other hand, for decades, estrogen-induced chemical castration has been used in the treatment of prostate cancer. Both ERα and ERβ are found in the prostate and the two types of ERs may have distinct, perhaps opposing effect on carcinogenesis. ENL binds ERs albeit with low affinity, and acts as a weak estrogen agonist in ERα- and ERβ-mediated transactivation, preferentially through ERα [47,48]. In ER binding and reporter gene assays, ENL has not shown any antiestrogenic action [48,49]. ENL bound to ER may cause a selective activation of estrogen response element (ERE)-regulated genes but it fails to activate non-classical SP-1 and AP-1 regulated estrogen responsive genes [47]. 

*In vivo* findings on estrogenicity of ENL have been contradictory. In immature mice, administration of ENL has not shown estrogen-like response in uterus [48,50,51]. However, in ERE-Luciferase reporter mice ENL had uterine-selective luciferase expression and induced a partial uterotrophic response suggesting that ENL is a partial estrogen agonist in the immature uterus [47]. In male mice, activation of ERE-Luciferase transgene expression has been observed in the ventral prostate in intact mice but not in castrated mice [47]. However, there is no evidence that lignans would act on hypothalamus-pituitary-testis axis and decrease testosterone concentrations and consequently decrease the growth-promoting effect of testosterone on the prostate. In conclusions, there is not enough evidence to argue that ENL is a full estrogen agonist. The concept that lignans are selective estrogen receptor modulators (SERMs) [48] is intriguing but needs to be confirmed by further experiments. 

Several additional mechanisms for lignan action in carcinogenesis have been suggested such as inhibition of 5α-reductase activity [52] and transactivation through human androgen receptor [53]. Many dietary plant lignans as well as enterolignans possess potent antioxidative activity *in vitro* [54,55,56] that may have significance in elimination of DNA damages caused by oxygen and nitrogen radicals. It is known that sesame seed lignans elevate blood tocopherol concentrations [57] suggesting one putative mechanism of dietary lignans in prostate carcinogenesis.

## 5. Surrogate Endpoints for Clinical Interventions

Adequately designed and well-conducted randomized clinical trials, targeted for cancer prevention and using the reduction of cancer incidence as a primary endpoint are long-lasting and expensive. The SELECT study serves as a good example [58]. The objective of SELECT was to determine whether selenium, vitamin E, or both could prevent prostate cancer and other diseases with little or no toxicity in relatively healthy men. Previously, secondary analyses of two randomized controlled trials and supportive epidemiologic and preclinical data had indicated the potential of selenium and vitamin E for preventing prostate cancer. Over 35,000 men were recruited in the SELECT study. They were randomly assigned to take one of four sets of supplements or placebos, with more than 8,000 men in each group. The median overall follow-up was over five years. The study concluded that prostate cancer was not prevented by selenium or vitamin E, alone or in combination [3]. The group's network consisted of more than 5,000 physician-researchers at nearly 550 institutions. SELECT was funded by NCI for $114 million, with additional funds from the National Center for Complementary and Alternative Medicine, and with sub-studies funded and conducted by the National Heart, Lung and Blood Institute, the National Institute of Aging and the National Eye Institute at NIH. 

Increasing knowledge of genetic predisposition, histopathological cancer precursors, and molecular basis of carcinogenesis allow the use of surrogate endpoints for demonstration of chemopreventive efficacy within a reasonable time and with affordable costs. Surrogate biomarkers should correlate with diminution or reversal of malignant potential and should have good predictivity for cancer prevention. It has been estimated that maximum three years intervention with several hundreds of subjects is sufficient for demonstrating primary prevention by using appropriate surrogate markers [59]. The most important criterion for a good surrogate biomarker in prostate cancer is the correlation of the marker alteration with the incidence of cancer [59]. 

Prostate specific antigen (PSA) is a standard serum biomarker used for screening of prostate cancer [60]. However, some doubts have been presented concerning the use of PSA as a surrogate for prostate cancer risk [61]. These include the limited specificity of PSA production e.g. in aging men, a major target group for prostate cancer chemoprevention. With advancing age PSA production is increased because of increasing prostatic volume or chronic nonbacterial prostatitis and other nonspecific factors. Therefore, other prognostic factors secondary to PSA need to be assessed when evaluating cancer risk [60]. In addition to age and prostate volume, ethnic group, family history, secondary prognostic factors include the presence of prostatic intraepithelial neoplasia (PIN).

In prostate cancer, both grade and extent of PIN are promising surrogate biomarkers for clinical chemopreventive studies. The prevention of PIN progression from mild to moderate or severe forms and to cancer, as well as the regression of PIN could be used to determine the chemopreventive efficacy of various substances. Currently, the detection of PIN is based on histological evaluation of biopsies obtained via random sampling. However, advances in imaging technologies are likely to offer new opportunities in diagnosis and biopsy sampling. Moreover, PIN evaluation may be combined with other surrogate biomarkers such as serum PSA (as described above) or tumor cell proliferation [62]. Proliferation (Ki-67 labelling index) has a significant prognostic value in assessing clinical progression and disease-specific mortality as it is considered as a marker of the Gleason grade. The Gleason grade is based on the glandular architecture of cancer that predicts the behavior of the carcinoma. Evaluation of PIN can also be combined with other surrogate biomarkers such as tumor microvessel density or apoptosis or molecular markers e.g. expression of androgen receptors, p53, b-3 integrin, or soluble vascular endothelial growth factor. 

## 6. Clinical Intervention Studies

Until now, only the effects of lignan-rich flaxseed on prostate cancer prevention have been studied both in primary and secondary prevention settings *i.e.* in patients without and with diagnosed cancer, respectively (Table 2). The effects of low-fat flaxseed supplemented diet on nonmalignant prostatic epithelium with PIN lesions were studied in men scheduled to undergo rebiopsy of prostate [63]. A significant decrease was observed in serum total PSA and prostatic epithelium proliferation. However, the low number of the lesions did not allow assessment of proliferation rate in PIN at follow-up. In the study, no change in serum total testosterone concentration was observed. 

**Table 2:** Summary of the intervention studies performed with lignan-rich flaxseed on prostate cancer.

*Study subjects*	*Intervention*	*n*	*Duration*	*Effects of intervention*	*Ref.*
Men with PIN scheduled repeated biopsies	Flaxseed 30 g/day combined with low-fat (≤20% of kcal) diet	15	6 months	Decreased serum total PSA and proliferation rate of benign epithelium.	[63]
PC patients awaiting prostatectomy	Flaxseed 30 g/day combined with low-fat (≤20% of kcal) diet	25	average 34 days	Significant decrease in total testosterone and free androgen indices. Among men with Gleason sums of ≤ 6 decreased tumor proliferation index. Increased tumor apoptotic scores in flaxseed group compared to historic controls.	[64]
PC patients awaiting for prostatectomy	Control (usual) diet	41	average 30 days	Significantly reduced tumor proliferation rates with flaxseed supplemented diets.	[65]
	Flaxseed 30 g/day diet	40			
	low-fat (≤ 20% of kcal) diet	40			
	Flaxseed 30 g/day combined with low-fat diet	40			

The effects of flaxseed supplemented diet on prostate cancer patients have been explored in two studies [64,65] (Table 2). In the first study [64], prostate cancer patients scheduled to undergo radical prostatectomy were on low-fat diet supplemented with 30 g of ground flaxseed for an average of 34 days. Among men with Gleason sums of ≤6 the proliferation index was reduced. Additionally, distribution of tumor apoptotic indices differed significantly between the historic control and flaxseed groups. The total testosterone, and free androgen index were decreased in serum while no significant effects on serum total PSA was observed. In the second study [65] (Table 2), the effect of flaxseed supplementation alone, low-fat diet alone, and combination of flaxseed supplementation with low-fat diet was compared with the control diet (usual diet) in prostate cancer patients scheduled for prostatectomy. In both flaxseed administered groups, the intervention period of 22-32 days resulted in significantly reduced tumor proliferation rates. No changes in tumor apoptosis or serum PSA, SHBG, testosterone, IGF-1, IGFBP-1, and C-reactive protein concentrations were observed.

These intervention studies indicate that lignan-rich diets may have potential to modulate prostate cancer risk associated biomarkers. Flaxseed may primarily inhibit proliferation of both benign prostatic epithelium as well as early stage prostate cancers. Although, flaxseed reduced serum total PSA prior to cancer diagnosis the obtained effects on PSA production in the presence of cancer as well as on serum androgen levels were controversial. In these studies, SHBG and IGFBP-3 concentrations were unaffected indicating that in men they are not the major targets for flaxseed effects. 

In general, the positive findings of the pilot studies performed so far should be interpreted with caution. The findings at their best indicate but do not prove the concept of prevention of prostate cancer with lignan-rich diets. The studies suffer mainly from a small sample size (a few tens of subjects at most) and various statistical problems. In some studies, outliers have been excluded from analysis which violates the intention-to-treat principle presuming analyses on all randomized subjects. It should also be noted that analysis of changes between two time points within one treatment group does not provide solid evidence of the intervention effects. Instead, comparisons between randomized treatment groups should be preferred to obtain direct evidence for the effects and to exclude the putative placebo effects. 

## 7. More Information is Needed For a Health Claim

Is there enough information to substantiate a health claim on the risk reduction of prostate cancer by lignans? Currently, the answer is no. In the EU countries, the practical (market-oriented) way to answer the question is to assess the available information according to the guidelines and opinions given by the European Food Safety Authority (EFSA). EU-wide rules on health claims were adopted in 2006 to ensure that any health claim made about foods or its constituents are clear, accurate, and substantiated by scientific evidence. Claims regarding reduction of disease risk (pursuant to Article 14 of EC regulation No 1924/2006) have to be examined by EFSA and approved by the Commission and Member States. The opinions published (for the first time in 2008) and guidelines given by EFSA provide information on the scientific substantiation of the proposed health claims (http://www.efsa.europa.eu). These opinions and guidelines should be followed also in lignan research.

One of the first published opinions of EFSA considered plant sterols and blood cholesterol. The expert panel concluded that a cause-effect relationship has been established between the consumption of plant sterols and lowering of LDL cholesterol, in a dose-dependent manner. This conclusion was supported by thirty randomized double-blind placebo controlled trials. LDL-cholesterol is a well recognized risk factor for coronary heart disease (CHD) but there are no studies demonstrating that plant sterols have impact on CHD morbidity or mortality. This decision of the panel demonstrated acceptance of the use of a surrogate biomarker (LDL cholesterol) in health claims. One would assume that this principle is applicable also to chemoprevention of prostate cancer.

On the basis of current knowledge dietary lignan doses required for inhibition of carcinogenesis (>1 mg/kg body weight) exceed those obtained from typical Western diet (few milligrams per day) [24]. The required lignan doses can be achieved by supplementing the diet with lignans or lignan-rich products. What is lacking is the knowledge regarding the anticancer effects of pure lignan compounds in prostate cancer. Target population for evaluating the effect of lignans would consist of men with diagnosed high-grade prostatic intraepithelial neoplasia (HGPIN), and scheduled for follow-up and rebiopsy because of HGPIN in the prostate. As long-term administration is needed, the safety requirements for such interventions are stringent. Thus far, HMR is the only dietary lignan tested for its safety. The data supports the conclusion that HMR can safely be used at the maximum dose of 50 mg/d for adults.

The main flaxseed lignan, secoisolarisiresinol diglucoside, and other lignans such as HMR and sesamin are available in sufficient quantities to allow treatment of considerable number of men for weeks. The strategy for applying surrogate endpoints in chemopreventive drug development has been described. However, no clinical intervention trials have been conducted so far with pure lignans. There are probably multiple causes for the negligence. First of all, there is the basic dilemma to give recommendations in terms of food components, such as plant derived lignans or diets (e.g., vegetarian diet). The failures in resolving the impact of dietary factors on the risk of prostate cancer have raised doubts about the potential of any specific food component including lignans. The negligence may reflect pessimism of investigating the disease with latency of many decades. Further, intervention studies may bear risks and may end up with results showing no effect. 

When the commercial exploitation has already started it is unlikely that industry-sponsored and conducted research would meet the requirements of EFSA or national authorities. A more neutral approach would involve establishing review, approval and public funding for research which would not compromise the quality of the work. It would be preferable to have international interdisciplinary inter-institutional research teams financially supported by intergovernmental bodies, such as The European Research Council. Is this possible? Converging forces – the expansion of globalization, the increasing ease of communication and the trend of “open innovation” are reshaping the working environment of public universities, industries, governments, and philanthropic organizations [66]. This opportunity should not be lost to inertia and inaction. 

Feasible, future studies could involve more than one lignan, preferably all three compounds (secoisolarisiresinol diglucoside, HMR, and sesamin) available in sufficient amounts. Next questions would then concern the significance of matrix of the lignan-rich foods and the pattern of consumption required to obtain the claimed effect. Recent experiences indicate that single-agent interventions, even in combinations, may be an ineffective approach to primary prevention of prostate cancer. Consumption of lignan-rich foods may increase consumption of other constituents (e.g. omega-3-fatty acids from flaxseed) that may account, at least in part, for the anticarcinogenic action. On the other hand, the food matrix may also contain substances which annihilate or attenuate the anticarcinogenic actions of lignans 

Urologists, essential collaborators in clinical studies have accepted phytochemicals as supplements to medicinal treatments of prostatic diseases such as lower urinary tract symptoms and chronic non-bacterial prostatitis. At present, interest in use of nutraceuticals is high among men, particularly among those with prostate cancer [67]. Therefore it can be concluded, that the time has come to investigate the potential effects of lignans, alone and in combinations, on the development of prostate cancer in well-conducted, adequately powered clinical studies. Interest in and appropriate funding for these collaborative studies would give an opportunity to address the question on the prevention and progression of prostate cancer, a major health problem in men.
